# Review of methods for handling confounding by cluster and informative cluster size in clustered data

**DOI:** 10.1002/sim.6277

**Published:** 2014-08-04

**Authors:** Shaun Seaman, Menelaos Pavlou, Andrew Copas

**Affiliations:** a MRC Biostatistics UnitCambridge, CB2 0SR, U.K.; b Department of Statistical Science, University College LondonLondon, WC1E 6BT, U.K.; c MRC Clinical Trials Unit at University College LondonLondon WC2B 6NH, U.K.

**Keywords:** conditional maximum likelihood, confounding by cluster, contextual effect, informative cluster size, poor man's method, within-cluster effect

## Abstract

Clustered data are common in medical research. Typically, one is interested in a regression model for the association between an outcome and covariates. Two complications that can arise when analysing clustered data are informative cluster size (ICS) and confounding by cluster (CBC). ICS and CBC mean that the outcome of a member given its covariates is associated with, respectively, the number of members in the cluster and the covariate values of other members in the cluster. Standard generalised linear mixed models for cluster-specific inference and standard generalised estimating equations for population-average inference assume, in general, the absence of ICS and CBC. Modifications of these approaches have been proposed to account for CBC or ICS. This article is a review of these methods. We express their assumptions in a common format, thus providing greater clarity about the assumptions that methods proposed for handling CBC make about ICS and vice versa, and about when different methods can be used in practice. We report relative efficiencies of methods where available, describe how methods are related, identify a previously unreported equivalence between two key methods, and propose some simple additional methods. Unnecessarily using a method that allows for ICS/CBC has an efficiency cost when ICS and CBC are absent. We review tools for identifying ICS/CBC. A strategy for analysis when CBC and ICS are suspected is demonstrated by examining the association between socio-economic deprivation and preterm neonatal death in Scotland.

## 1. Introduction

Clustered data commonly arise in epidemiology, for example, patients clustered within hospitals, pupils within schools, and teeth within patients. Generalised linear mixed models (GLMM) [1] and generalised estimating equations (GEE) [2] are commonly used to analyse clustered data when interest is in the association between outcome *Y* and covariate vector ***X*** measured on each member of the cluster. GLMM give cluster-specific inference; GEE give population-average inference. Two issues in the analysis of clustered data are confounding by cluster (CBC) [3–9] and informative cluster size (ICS) [10–17].

Standard GLMM assume that the random effect ***u*** associated with cluster is independent of ***X*** values in the members of that cluster. Violation of this assumption has been called CBC, because even if there is no confounding within clusters, association of ***u*** with ***X*** means that there may be confounding in the population as a whole. An example is the association between childhood IQ (*Y*) and birth weight (***X***), with clusters being families [4]. Although many studies have found that heavier babies tend to have higher IQ, this may be due to confounding by complex family-level social and economic factors. Although one could adjust for some measure of familial socio-economic status, it is likely that such a measure would capture only some of the confounding. When there is CBC, each member's outcome *Y* depends on the ***X*** values of the other members in the same cluster. Covariate ***X*** is then said to have a ‘contextual effect’ [4]. Like standard GLMM, standard GEE assume that there is no contextual effect [18].

ICS refers to the situation where cluster size *N* varies and *Y* is not independent of *N* given ***X***. An example is a toxicology experiment in which pregnant dams are randomised to exposure (*X* = 1) or non-exposure (*X* = 0) to a toxicant and the presence (*Y* = 1) or absence (*Y* = 0) of birth defects in each of her *N* pups is noted [10]. Dams that are particularly susceptible to the effects of a toxicant may produce a higher proportion of pups with birth defects and simultaneously experience more foetal resorptions (and so have smaller litter sizes). Consequently, *Y* may be positively associated with *X* and negatively associated with *N* given *X*. Nevalainen *et al.* (and references therein) give other examples and discuss data-generating mechanisms giving rise to ICS [19]. Standard GLMM and GEE assume that cluster size is non-informative.

In some studies, missing data may be the reason for variation in cluster size. For example, in a cohort study involving *M* waves, an individual is a cluster, a set of measurements on that individual at a particular wave is a member, and *N* is the number of waves attended before dropout. In this case, interest may be in the association between *Y* and ***X*** in ‘complete clusters’, that is, the clusters composed of both the *N* observed members (before dropout) and the *M* − *N* missing members (after dropout), and inference about this association achieved by making some assumption about the missing data, for example, missing at random. We emphasise that we are not considering inference about such ‘complete clusters’. Instead, we are assuming that either there are no missing data or interest is in the *Y*–***X*** association in the clusters of observed members.

Several modifications of standard GLMM and GEE have been proposed for handling CBC and/or ICS. Most of the GLMM-based methods separate the effect of ***X*** into ‘within-cluster’ and ‘between-cluster’ components and focus on the former. These components differ when there is CBC [5], and the danger of using between-cluster estimates to describe within-cluster effects, commonly referred to as the ‘ecological fallacy’, has been known for many years [20,21]. Some methods estimate contextual effects in addition to the within-cluster effect, allow the within-cluster effect to vary from one cluster to another, or explicitly model the association between *Y* and *N*. Among the GEE-based methods are those that use the independence working correlation and weights based on *N* or ***X***. They vary in their target of inference.

When methods have been described for handling CBC, it has often been carried out without explicit reference to the potential problem of ICS and vice versa. Yet clustered data may be subject to both CBC and ICS simultaneously. In this article, we compare the various methods proposed for handling CBC and/or ICS, placing particular focus on the following: (i) the assumptions they require about both CBC and ICS; (ii) the relations between the methods; (iii) their estimands; and (iv) their relative efficiencies. We also propose some simple obvious additions to the available repertoire of methods. We summarise previous findings about when the standard GLMM consistently estimates within-cluster effects despite ICS and discuss when it may be appropriate to handle ICS by including *N* among the covariates ***X***. We also present a general discussion of choice of method and an illustrative analysis of clustered data potentially subject to CBC and ICS. The structure is as follows. In Section 2, we define ICS and CBC. Methods for cluster-specific and population-average inference are reviewed in Sections 3 and 4, respectively. Section 5 discusses the choice of method, and Section 6 illustrates the problem of CBC using data on infant mortality.

## 2. Informative cluster size and confounding by cluster

For a given cluster, let *N* denote its size, and let ***X***_*j*_ and *Y*_*j*_ denote the covariate vector and outcome, respectively, of the *j*th member of the cluster. Partition ***X*** as 
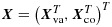
, where ***X***_va_ and ***X***_co_ are the cluster-varying and cluster-constant elements of ***X***, respectively. Assume that ***X***_co_ includes an intercept element, that is, an element that equals 1 for all members. Let X^*^=(***X***_1_,…,***X***_*N*_)^*T*^ and Y^*^=(*Y*_1_,…,*Y*_*N*_)^*T*^. So, X^*^ and Y^*^ contain covariates and outcome, respectively, for all members of the cluster. Let *H* be a random variable uniformly distributed on {1,…,*N*} and independent of X^*^ and Y^*^ given *N*. So, ***X***_*H*_ and *Y*_*H*_ are the covariates and outcome of a random member of the cluster. We use *f*(.) to denote a probability distribution.

Different but closely related definitions of ICS have been given in the literature. Dunson *et al.* [12], Gueorguieva [13], Chen *et al.* [17], and Neuhaus and McCulloch [16] considered random-effects models and said that cluster size is informative if the cluster-specific random effect ***u*** is not independent of *N*. Hoffman *et al.* [10], Williamson *et al.* [11] and Benhin *et al.* [14] considered marginal models and defined non-ICS to mean *E*(*Y*_*H*_∣***X***_*H*_=***x***,*N*) = *E*(*Y*_*H*_∣***X***_*H*_=***x***) for all ***x*** and ICS to mean that this equality does not hold. ICS according to the former definition implies ICS according to the latter definition but not vice versa. In particular, the latter definition does not require a random-effects model. A further definition is given by Nevalainen *et al.* [19], but we shall not need this here.

In the literature on random-effect models, the term ‘CBC’ has been used to mean that ***u*** is not independent of X^*^ given *N* [6,7,9]. It has been used less in the GEE literature, but essentially the same problem has been discussed there [3,15,18]. When all clusters are of the same size, CBC can be taken to mean that the expectation of *Y*_*j*_ given X^*^ depends on ***X***_*k*_*k* ≠ *j* as well as ***X***_*j*_ (i.e. *E*(*Y*_*j*_∣X^*^) ≠ *E*(*Y*_*j*_∣***X***_*j*_)). Pepe and Anderson [18] noted that GEE provide inconsistent estimation when this is the case, unless the independence working correlation is used. This definition of CBC is problematic when *N* varies, because *Y*_*j*_ is only defined in clusters with 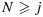. More generally, we interpret CBC to mean *E*(*Y*_*H*_∣X^*^) ≠ *E*(*Y*_*H*_∣***X***_*H*_). Huang and Leroux [15] used the term ‘informative covariate distribution’. Although they did not formally define this, they seem to mean *E*(*Y*_*H*_∣X^*^) ≠ *E*(*Y*_*H*_∣***X***_*H*_), and hence be talking about CBC.

Note that whether there is ICS or CBC may depend on which variables are included in ***X***. If *N* is included in ***X***, cluster size is automatically non-informative. Likewise, it may be possible to eliminate (or at least reduce) CBC by including observed cluster-level confounders in ***X***. As Williamson *et al.* [11] noted, if ***X*** is not cluster-size balanced, that is, if it is not true that *f*(***X***_*H*_∣*N*) = *f*(***X***_*H*_), then ICS induces CBC in general: the association between *Y*_*H*_ and ***X***_*H*_ is confounded by *N*.

## 3. Cluster-specific inference

### 3.1. Model and assumptions

Assume that


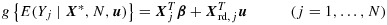
(1)

where *g* is a known link function, ***β*** is a vector of unknown parameters, ***X***_rd_ is a subvector of ***X*** that includes the intercept term, and ***u*** is an unobserved cluster-constant variable. We say that elements of ***X*** that are part of ***X***_rd_ ‘have random effects’, that the remaining elements of ***X*** ‘have fixed effects’, and that ***u*** is the random effect associated with ***X***_rd_. Partition ***β*** as 
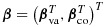
, where ***β***_va_ and ***β***_co_ are the elements of ***β*** corresponding to ***X***_va_ and ***X***_co_, respectively. Then partition ***β***_va_ as 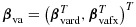, where ***β***_vard_ and ***β***_vafx_ denote the subvectors of ***β***_va_ corresponding to elements of ***X***_va_ that have random effects and fixed effects, respectively. Similarly, let ***u***_va_ denote the subvector of ***u*** corresponding to cluster-varying elements of ***X***_rd_. Assume that if ***u***_va_ is not empty, then it is independent of X^*^ given *N*.

Equation (1) implies that any dependence of *Y*_*j*_ on the values ***X***_va,*k*_*k* ≠ *j* of other members of the cluster must be through a cluster-level summary (e.g. mean) of ***X***_va,1_,…,***X***_va,*N*_ and the effect of this summary is the same for all members *j* and is absorbed into ***u***. Likewise, any dependence on *N* is also absorbed into ***u***. Note that because ***u*** includes a random intercept, ***β***_co_ is not identifiable unless further assumptions are made about ***u***.

Several statistical methods are based on Equation (1). Before describing these, we list further assumptions that could be made about the joint distribution of (*N*,X^*^,Y^*^,***u***). Assumptions A1 and A2 together define GLMM. A3 and A4 are the non-CBC and non-ICS assumptions, respectively. Assumptions A5, A6, and A7 will be used less frequently than A1–A4 in this article. They are needed in Sections 3.3.1, 3.3.7, and 3.3.8, respectively.

(A1) *Y*_1_,…,*Y*_*N*_ are conditionally independent given X^*^, ***u*** and *N*; and *f*(*y*_*j*_∣X^*^,*N*,***u***)= exp[{*y*_*j*_*θ*_*j*_−*b*(*θ*_*j*_)}/*a*(*φ*) + *c*(*y*_*j*_,*φ*)], where *a*(.), *b*(.) and *c*(.) are known functions, *φ* is the scale parameter, and 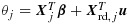. For ease of presentation, we have assumed a canonical link function, but this is not necessary.
(A2) ***u*** is independent of X^*^ and *N*, and has a specified distribution (usually normal) with mean zero.
(A3) ***u*** is conditionally independent of X^*^ given *N*.
(A4) ***u*** is independent of *N*.
(A5)  Either ***X*** is cluster constant or ***X***_1_,…,***X***_*N*_ are independent and identically distributed (i.i.d.) given *N*. Furthermore, the distribution of ***X*** given *N* does not depend on *N* (and so ***X*** is cluster-size balanced).
(A6) ***u***_va_ is independent of *N* and is normally distributed with mean zero.
(A7) ***u*** is normally distributed with mean zero. ***X***_co_ is independent of ***u***. The distribution of *N* given ***u*** and ***X***_co_ takes a specified form. If ***X***_va_ is not empty, then conditional on ***X***_co_, *N* and ***u***, variables ***X***_va,1_,…,***X***_va,*N*_ are i.i.d. with a distribution that does not depend on *N* or ***u***. (This allows for ICS but rules out CBC).

A2 implies A3 and A4. If ***u*** is normally distributed, A2 also implies A6.

### 3.2. Interpretation of model parameters

Here we briefly discuss interpretation of ***β*** and Var(***u***). Seaman *et al.* [22] discussed it in more depth. If A3 and A4 hold, interpretation is unproblematic. ***β***_vafx_ can be interpreted in terms of a within-cluster comparison. That is, if two members of the same cluster have covariates ***X***_*j*_ and ***X***_*k*_ that differ only in elements corresponding to ***β***_vafx_ (i.e. in cluster-varying elements with fixed effects), then the difference between their expected values of *Y* is ***β***^*T*^(***X***_*j*_−***X***_*k*_) for a linear mixed model (LMM). For GLMM more generally, the expected values are transformed by the link function *g*. For example, for logit link, ***β***^*T*^(***X***_*j*_−***X***_*k*_) is the log odds ratio of *Y* for the two members. ***β***_vard_ and Var(***u***_va_) can be interpreted as the mean and variance over clusters for such within-cluster comparisons. Elements of ***β***_co_ can be interpreted in terms of between-cluster comparisons. That is, if two members belonging to different clusters have covariate values ***X***_*j*_ and ***X***_*k*_ that differ only in ***X***_co_, then the difference between their expected *Y* values is 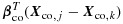 for an LMM and, more generally, 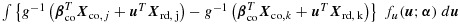 for GLMM. Causal interpretations are also possible if additional assumptions are made [22].

Even if A3 or A4 does not hold, ***β***_vafx_ can still be interpreted, as in the preceding paragraph, in terms of a within-cluster comparison. We assume throughout Section 3 that ***u***_va_ is independent of X^*^ given *N*. Therefore, if A6 holds, ***u***_va_ is independent of X^*^ and *N*, and so ***β***_vard_ and Var(***u***_va_) can be interpreted, as in the preceding paragraph, as the mean and variance for within-cluster comparisons. If A7 holds, ***β***_co_ can be interpreted, as in the preceding paragraph, in terms of a between-cluster comparison, and if 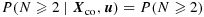, then ***β***_vard_ and Var(***u***_va_) are the mean and variance for within-cluster comparisons [22].

### 3.3. Estimation methods

Methods for obtaining cluster-specific inference are now described. Table I summarises the assumptions they require and the quantities they estimate.

**Table I:** Methods for cluster-specific inference: estimands and assumptions needed to estimate them consistently. See main text for more details.

	Assumptions	Estimands	Notes
*Methods assuming non-ICS and no CBC*			
GLMM	A1, A2	***β***, Var(***u***)	Alternative assumptions for LMM:
			A3 and A4 for ***β***; or A3, A5 and
			***X***_rd_=1 for non-intercept elements
			of ***β***
*Methods allowing ICS and CBC but requiring****X***_rd_=1			
Conditional ML 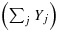	A1	***β***_va_	A1 not needed for LMM
Poor man's method	A1	***β***_va_, context effects	Same as conditional ML for LMM;
			approximate otherwise
Brumback *et al.'s* method	A1	***β***_va_, context effects	Generalisation of poor man's method
Conditional GEE	None	***β***_va_	Only for identity/log link
*Methods allowing ICS and/or CBC, and not requiring****X***_rd_=1			
Conditional ML 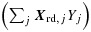	A1	***β***_vafx_	
Model expectation	A1	***β***_va_, context effects	Generalisation of Brumback's method
of random intercept			
Treat random intercept	A1. Also:	***β***_va_,	Same as conditional ML if ***X***_rd_=1
as fixed effect	A6 if ***X***_rd_≠1;	Var(***u***_va_)	and identity/log link; same as conditional
	and *N* large		LMM if ***X***_rd_≠1 and iden. link
	if not LMM		
Joint model *Y* and *N*	A1, A7	***β***, Var(***u***)	See 3.3.8 for alternative to A7 when
			***X***_rd_=1

#### 3.3.1. Maximum likelihood estimation of generalised linear mixed models

Fitting the GLMM defined by Equation (1), A1 and A2 by maximum likelihood (ML) or restricted ML consistently estimates ***β*** and Var(***u***) when these assumptions are satisfied. However, consistent or approximately unbiased estimation is also possible under weaker conditions.

For the LMM, A3 and A4 together with *E*(***u***) = 0 are sufficient for consistent estimation of ***β***. This is because Equation (1) then implies 
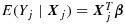
, and the LMM's score equations for ***β*** can be viewed as GEE with a particular choice of working correlation structure. If A1 also holds, Var(***u***) is consistently estimated [23].

Many researchers have investigated how important A2 is for non-LMM GLMM when A1, A3 and A4 hold and *E*(***u***) = **0**. McCulloch and Neuhaus [24] reviewed the evidence. They conclude that when ***X***_rd_=1, deviations from A2 cause only minimal bias in the ML estimator of the non-intercept elements of ***β*** (particularly for cluster-varying covariates whose mean is constant between clusters), but there can be some loss of efficiency. Bias in the ML estimator of the intercept element of ***β*** may be considerable. Neuhaus *et al.* [25] considered the situation where ***X***_rd_≠1. They found little bias for non-intercept elements of ***β*** whose corresponding elements of ***X*** are orthogonal to ***X***_rd_. Bias in ***β***_vard_ also tended to be small in most cases. Bias in the estimated variance of the random effects could be much greater.

The importance of A3 for consistent estimation of ***β*** has been demonstrated by Heagerty and Kurland [26] and Neuhaus and McCulloch [6].

Neuhaus and McCulloch [16] investigated whether A4 is necessary when ***X***_rd_=1. For an LMM with ***X***_rd_=1, they proved that when A3 and A5 hold, the ML estimators of non-intercept elements of ***β*** are consistent even when A4 does not hold. They also proved that the same is true for non-LMM GLMM when the non-intercept elements of ***β*** equal zero, and found, in a simulation study, that bias in the ML estimators of the non-intercept elements of ***β*** was small even when these elements were non-zero. When ***X***_rd_≠1, the ML estimators of non-intercept elements of ***β*** are inconsistent in general [16] for both LMM and GLMM. Su *et al.* [27] demonstrated that the bias in the subvector of ***β*** corresponding to ***X***_rd_ can be large.

#### 3.3.2. Conditional maximum likelihood

When A1 holds and *g* is the canonical link, the conditional distribution of Y^*^ given X^*^, *N*, and 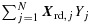 does not involve ***β***_co_, ***β***_vard_ or parameters of Var(***u***). So, conditioning on 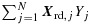 eliminates those parameters from the likelihood, leaving only ***β***_vafx_ [28]. Maximising the resulting conditional likelihood yields the conditional ML estimator, which is a consistent estimator of ***β***_vafx_. For example, when *Y* is discrete, the contribution of a cluster to the conditional likelihood is 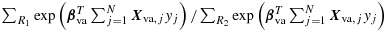, where *R*_1_ and *R*_2_ are the sets of all possible values for (*y*_1_,…,*y*_*N*_) such that, respectively, 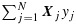 equals its observed value and 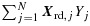 equals its observed value [29]. Verbeke *et al.* [30] gave the form of the conditional likelihood for the LMM. Conditional ML is easy to apply in standard software (Appendix A). It is most often used when ***X***_rd_=1, so that conditioning is on 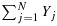. When ***X***_rd_=1 and *g* is the identity link, A1 is not necessary for consistent estimation of ***β***_va_ (Appendix B).

#### 3.3.3. Poor man's method

When there is only one covariate and it is cluster-varying and has fixed effect (so, ***X***_co_=***X***_rd_=1 and ***X***_va_ is a scalar), an alternative to conditional ML is the ‘poor man's' method [31]. In this method, a modified form of the GLMM with normally distributed random intercept is fitted by maximum likelihood. The modification is that *X*_va,*j*_ is replaced by 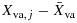 and 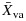, where 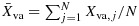. The parameters *γ*_1_ and *γ*_2_, say, associated with 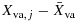 and 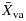 are called, respectively, the ‘within-cluster’ and ‘between-cluster’ effects of *X*_va_ [31]. The term ‘contextual effect’ has been used variously to mean *γ*_2_−*γ*_1_ or *γ*_2_ [32–34]. If A3 holds, *γ*_1_ and *γ*_2_ both equal *β*_va_ in Equation (1); otherwise, only *γ*_1_ equals *β*_va_. Whereas the original unmodified GLMM assumes A3 and uses both within-cluster and between-cluster comparisons of *Y* to estimate the common effect, the poor man's method aims to estimate *β*_va_ using only within-cluster comparisons.

In the case of the LMM, *γ*_2_ can be interpreted as the true slope in a linear regression of 
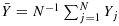
 on 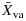. Begg and Paredis [4] suggested that *γ*_2_−*γ*_1_ is more easily interpreted than *γ*_2_: *γ*_2_−*γ*_1_ describes the difference between the (*g*-transformed) expected outcome of two members with the same *X*_va_ belonging to clusters with the same *u* but different 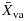, whereas *γ*_2_ describes the difference for two members with the same deviation 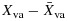 from their cluster means. For this reason, they advocated fitting the reparameterised version of the poor man's model 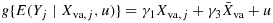, where *γ*_3_=*γ*_2_−*γ*_1_.

When *g* is the identity link, the ML estimate of the parameter associated with 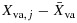 and its corresponding estimated standard error are identical to the conditional ML estimate and its corresponding estimated standard error. Curiously, this result does not seem to be known. Neuhaus and Kalbfleisch [31] found a small difference (≈1% of a SE) between the estimates from conditional ML and the poor man's method when analysing one specific data set with a single covariate. They described these two estimates as ‘nearly identical’. Neuhaus and McCulloch [6] and Goetgeluk and Vansteelandt [7] also referred to the result of this analysis and used the same phrase: ‘nearly identical’. We are not aware of any published proof that the methods are, in fact, equivalent and so have provided one in Appendix C.

Neuhaus and McCulloch [6] and Brumback *et al.* [9] studied the poor man's method for binary *Y*. In simulations, they found the bias for *β*_va_ was small. However, Brumback *et al.* [35] demonstrated that bias could be more substantial. For Poisson-distributed *Y*, Goetgeluk and Vansteelandt [7] investigated a population-average version of the poor man's method (Section 4.1.3). The maximum bias they found in simulations was 25%, and the minimum coverage of the nominal 95% confidence interval was 89%.

#### 3.3.4. Method of Brumback *et al*

Brumback *et al.* [9] pointed out that if ***X***_co_=***X***_rd_=1 and ***X***_va_ is a scalar and the random intercept *u* can be written in the form 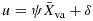, where *ψ* is an unknown parameter and 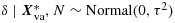, then 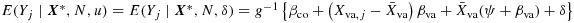, and hence the poor man's model is correctly specified. Thus, when the random intercept has a normal distribution with mean linearly related to 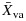 and variance independent of 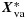, the poor man's method gives consistent estimation of ***β***. Note that this argument requires A1 and A4.

Brumback *et al.* [9] noted that *u* might not depend linearly on 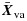 and proposed a more general method. This involves specifying a model 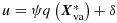, where *q* is a known function and 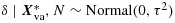. The GLMM with 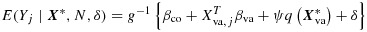 would then be fitted. If the model for *u* is correctly specified and A1 holds, the ML estimator of ***β*** is consistent. The poor man's method is a special case of this approach: it uses 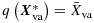. Brumback *et al.* illustrated their method with 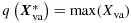.

#### 3.3.5. Modelling expectation of random intercept

The following generalisation of the method of Brumback *et al.* (and hence of the poor man's method) can be used to deal with CBC and/or ICS and allows ***X***_co_≠1, ***X***_rd_≠1 and vector ***X***_va_. It involves modelling the conditional expectation of the random intercept given X^*^ and *N*. In this section only, we shall write 
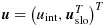
, where *u*_int_ is the random intercept (‘slo’ means ‘slope’), and denote the subvector of ***X***_rd_ composed of all but its intercept element by ***X***_rdslo_.

Specify a model 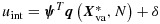, where ***q*** is a known function, ***ψ*** is an unknown parameter, and (*δ*,***u***_slo_)^*T*^∣X^*^,*N* ∼ Normal(**0**,**Σ**). Then Equation (1) can be written as 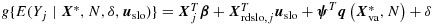. If this GLMM is fitted, the ML estimator of ***β*** will be consistent, provided that the model for *u*_int_ is correctly specified and A1 holds.

To deal with CBC, one might use, for example, 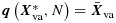 or 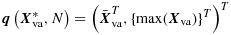. To deal with ICS, one might use, for example, 
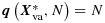
 or 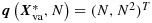. To deal simultaneously with CBC and ICS, one might use, for example, 
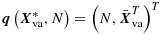
 or 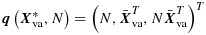. Such inclusion of *N* as a covariate in the GLMM, and possibly of interactions between *N* and elements of ***X***, would be natural if there were scientific interest in the effect of *N*. For example, one might be interested in the effect of the numbers of patients (*N*) treated by a surgeon on his or her operational success rate or how the effect on that success rate of participation in a surgeons' training programme (*X*) depends on the number of patients [36]. Likewise, including 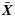 as a covariate might be useful if there were interest in contextual effects.

#### 3.3.6. Conditional generalised estimating equations

Goetgeluk and Vansteelandt [7] proposed conditional GEE for estimating ***β***_va_ when ***X***_rd_=1 and *g* is the identity or log link function. For the identity link, the conditional ML score equations are a special case of conditional GEE (Appendix C). Whereas the former are derived under the assumption that, given X^*^ and ***u***, *Y*_1_,…,*Y*_*N*_ are independent with equal variance, the latter allow *Y*_1_,…,*Y*_*N*_ to be correlated and/or have different variances, and estimate these correlations and variances from the data. It is unclear what the efficiency cost of this estimation will be when the assumption holds. When the assumption is violated, conditional ML is still consistent, but conditional GEE may then be more efficient. For the log link, conditional GEE have the advantage that it does not require A1 to hold for consistent estimation, whereas conditional ML does. Software for applying conditional GEE is not readily available.

#### 3.3.7. Treating the random intercept as a fixed effect

If A1 holds and either ***X***_rd_=1 or A6 holds, then one approach to estimating ***β***_va_ and Var(***u***_va_) is to treat the random intercept as a fixed effect to be estimated (and remove from Equation (1) 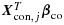 and all cluster-constant elements of ***X***_rd_ and corresponding elements of ***u***, in order to avoid parameter non-identifiability). Equation (1) and A1 then describe a generalised linear model (if ***X***_rd_=1) or GLMM (if ***X***_rd_≠1) with cluster included as a categorical variable.

The ML estimator from this model is not, in general, consistent, because the number of parameters increases with the number of clusters [37]. However, when clusters are large, it may be approximately unbiased. For binary *Y*, the so-called rule of 10 advocates that the mean number of events per cluster 
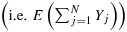
 should exceed 10 [38].

In the following special cases, the ML estimator is consistent. When ***X***_rd_=1 and the model is a linear or Poisson regression, the ML estimator of ***β***_va_ is identical to the conditional ML estimator [30,39]. When ***X***_rd_≠1, *Y* is normally distributed, *g* is the identity link, and A1 and A6 hold, treating the random intercept as a fixed effect is equivalent to fitting what Verbeke *et al.* called a ‘conditional LMM’ and gives consistent estimation of ***β***_va_ and Var(***u***_va_) [30].

When the number of clusters is large, fitting a model containing a separate parameter for each cluster can be computationally intensive. For normally distributed *Y*, Verbeke *et al.* [30] describe alternative, less intensive algorithms (Appendix A).

#### 3.3.8. Joint modelling of *Y* and *N*

The GLMM method requires A4 for consistent estimation. This assumption can be avoided by combining the GLMM with a model for the distribution of *N* given ***u*** and ***X***_co_. Such a joint model for *Y* and *N* is known as a shared-parameter model in the missing-data literature [40]. Dunson *et al.* [12] and Gueorguieva [13] adopted this approach, which Seaman *et al.* [22] discussed in detail. When A1 and A7 hold, the joint model provides consistent estimation of ***β*** and Var(***u***) [22]. It can be fitted in sas [13].

Chen *et al.* [17] also used the joint model. They assumed A1, ***X***_rd_=1, and that the distribution of *N* given *u* and ***X***_co_ does not depend on ***X***_co_, but modified A7 by allowing ***X***_va_ to be a deterministic function of *j* (e.g. ***X***_va,*j*_=*j*). This method gives consistent estimation of ***β***_va_ [22]. However, because there are other, simpler methods available for estimating ***β***_va_ when ***X***_rd_=1, this method may be of limited interest unless one wishes also to model *N*, *u*, and the effect of ***X***_co_.

## 4. Population-average inference

Cluster-specific inference is about the effect of ***X*** on *Y* conditional on cluster size *N* and cluster-level latent variable ***u***. Population-average inference, on the other hand, concerns the effect of ***X*** unconditional on *N* or any latent variables (although if desired, *N* can be conditioned on by including it in ***X***). One way to make such inference is to fit GLMM and then integrate out ***u***. However, GEE-based approaches are more commonly used. In this section, we examine methods for population-average inference. Unlike in Section 3, where the meaning of cluster-specific inference is always defined by Equation (1), the meaning of ‘population-average inference’ changes according to the method used.

Let *e*_*T*_(***x***) = *E*(*Y*_*H*_∣***X***_*H*_=***x***) and *e*_*A*_(***x***) = *E*(*NY*_*H*_∣***X***_*H*_=***x***)/*E*(*N*∣***X***_*H*_=***x***) (*H* was defined in Section 2). The quantity *e*_*T*_(***x***) describes the relation between *Y* and ***X*** in a randomly chosen member of a randomly chosen cluster. Inference about *e*_*T*_(***x***) is ‘inference about a typical member of a typical cluster’ [11]. Inference about *e*_*A*_(***x***) concerns the relation between *Y* and ***X*** among all members. Clusters contribute to this ‘inference about all members’ in proportion to their size. If cluster size is non-informative, *e*_*T*_(***x***) = *e*_*A*_(***x***); otherwise, they differ in general. As discussed by Seaman *et al.* [22], either may be of interest. Many of the methods for population-average inference assume either that 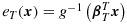 or that 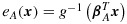, where *g* is a known link and ***β***_*T*_ and ***β***_*A*_ are unknown parameters, and some require the following assumption.

(A8) *E*(*Y*_*j*_∣***X***_*j*_=***x***,X^*^,*N*) = *E*(*Y*_*j*_∣***X***_*j*_=***x***,*N*) = *E*(*Y*_1_∣***X***_1_=***x***)for all ***x***and *j*≤*N*. That is, the expectation of *Y*_*j*_given X^*^and *N* depends only on ***X***_*j*_and is the same for all *j*. (This implies non-ICS and no CBC, and so *e*_*T*_(***x***) = *e*_*A*_(***x***).)

### 4.1. Methods

Methods for population-average inference are now described, with their targets of inference and the assumptions they require. Table II provides a summary.

**Table II:** Methods for population-average inference: estimands and assumptions needed to estimate them consistently. See main text for further detail.

Method	Assumptions	Estimand	Notes
*Methods assuming non-ICS and no CBC*			
GEE	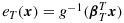 , A8	*e*_*T*_(***x***)	*e*_*A*_(***x***) = *e*_*T*_(***x***)
Marginalise over ***u*** in	GLMM correct (so A8	*e*_*T*_(***x***)	*e*_*A*_(***x***) = *e*_*T*_(***x***)
GLMM	and 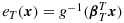 )		
*Methods assuming no CBC*			
Marginalise over ***u*** in	Joint model correct	*e*_*T*_(***x***)	
joint model			
*Methods allowing ICS and/or CBC*			
IEE	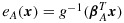	*e*_*A*_(***x***)	*e*_*T*_(***x***) = *e*_*A*_(***x***) when A8
Poor man's method	*E*(*Y*_*j*_|X^*^,*N*) depends	See text	
	only on ***X***_*j*_ and 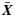		
Include *N* as covariate	Model for *E*(*Y*_*j*_|***X***_*j*_,*N*)	See text	
Weighted IEE	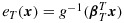	*e*_*T*_(***x***)	Useful when cluster-size
			balance or *N* on causal pathway
Type 1 doubly weighted	Each cluster has all	See text	
IEE	possible ***x***		
Type 3 doubly weighted	Propensity score model	Marginal treatment effect	Method of Joffe *et al.* [41] with
IEE		in typical member	extra weighting by *N*^−1^

#### 4.1.1. Marginalising over ***u*** in generalised linear mixed models

The assumptions (Equation (1), A1, and A2) of the GLMM imply A8, and so *e*_*T*_(***x***) = *e*_*A*_(***x***). If the assumptions of the GLMM hold, *e*_*T*_(***x***) can be estimated by fitting the GLMM and then marginalising over the distribution of ***u***. If, furthermore, *g* is the identity link, then *e*_*T*_(***x***) = ***β***^*T*^***x***, where ***β*** is defined by Equation (1), and so there is no need actually to perform this marginalisation. Similarly, if *Y* is binary, ***X***_rd_=1 and *u* has a bridge distribution with parameter *φ* (e.g. for the logit link function, *u* has density (2*π*)^−1^sin(*φu*)/{cosh(*φu*) + cos(*φu*)}, −*∞* < *u* < *∞*, 0 < *φ* < 1), then *e*_*T*_(***x***) = *φ****β***^*T*^***x*** [42]. In other cases, *e*_*T*_(***x***) is not, in general, a simple parametric function of ***x***, but marginalisation could be achieved by numerical integration for specific values of ***x***.

Likewise, when the assumptions (Equation (1), A1, and A7) of the joint model are satisfied, *e*_*T*_(***x***) (≠*e*_*A*_(***x***)) could be estimated by fitting the joint model and marginalising over the random effects.

#### 4.1.2. Independence estimating equations and generalised estimating equations

The GEE with independence working correlation (independence estimating equations (IEE)) consistently estimate ***β***_*A*_ when 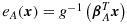 holds [22]. GEE with non-independence working correlation consistently estimate ***β***_*A*_ when 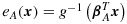 and A8 holds [18]. When A8 is violated, the value to which the GEE estimator of ***β***_*A*_ converges as the number of clusters tends to infinity depends on the choice of working correlation, and equals neither ***β***_*A*_ nor ***β***_*T*_ in general [11,18]. So, when there is ICS or CBC, GEE should not be used with a non-independence working correlation.

#### 4.1.3. Poor man's generalised estimating equations method

Berlin *et al.* [3] describe a GEE-based version of the poor man's method. They described it for a single covariate, but here we generalise to allow for multiple covariates. This method assumes that any dependence of *Y*_*j*_ on ***X***_*k*_*k* ≠ *j* and *N* is mediated through the mean of ***X*** in the cluster, that is, 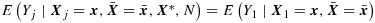 for all *j*≤*N*, ***x*** and 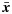, and that 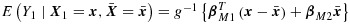. Parameters ***β***_*M*1_ and ***β***_*M*2_ are estimated using GEE. ***β***_*M*1_ would be interpreted as the difference in the expected outcome of two members with values of ***X*** differing by one unit but with the same cluster mean. Note that (i) ***β***_*M*1_ cannot be interpreted causally, because intervening to change ***X*** would also change 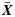 [7,32]; and (ii) 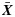 could be replaced by another function of X^*^, just as Brumback *et al.* [9] did with the GLMM (Section 3.3.4).

#### 4.1.4. Including cluster size as a covariate

In Section 3.3.5, we observed that cluster size could be made non-informative by including *N* as a covariate in GLMM. This would not change the interpretation of ***β***_va_ as the within-cluster effect of ***X***_va_. One could also eliminate ICS in GEE by including *N* as a covariate. In general, however, this changes the meaning of the parameter for ***X***: it now describes the association of *Y* and ***X*** conditional on *N*. As discussed in Section 3.3.5, it would be natural to include *N* if there were scientific interest in the effect of *N*. However, there are at least three potential reasons why one might not want to include *N* as a covariate.

First, one may wish to estimate the overall effect of ***X*** on *Y* averaged over all clusters, rather than the effect conditional on *N*. When ***X*** is cluster-size balanced, this could be achieved by estimating the effect of ***X*** on *Y* in clusters of each size *N* separately and averaging these effects. When, however, ***X*** is not cluster-size balanced, this simple approach is not possible without specifying a model for *f*(***X***_*H*_∣*N*). Moreover, even when ***X*** is cluster-size balanced, there may be an issue of ‘non-collapsibility’: if *g*{*E*(*Y*∣***X***,*N*)} is a linear function of ***X*** and *N*, this does not usually imply an equally simple functional form for *E*(*Y*∣***X***) unless *g* is the identity link. Note that ICS does not always cause the association of *Y* and ***X*** conditional on *N* to differ from the marginal association. If ***X*** is cluster-size balanced and 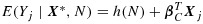, for some function *h*(*N*) of *N*, then 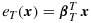, 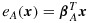, and the non-intercept elements of ***β***_*T*_, ***β***_*A*_ and ***β***_*C*_ are equal.

Second, *N* may lie on the causal pathway from ***X*** to *Y*. Consider, for example, a toxicology trial in which pregnant mice are randomised to exposure to a toxin or no exposure [12]. Here, clusters are litters, members pups, *N* the litter size, *X* an indicator of exposure of the mother, and *Y* the weight of a pup. The toxin may cause foetal resorptions, in which case exposed mothers tend to have smaller litters. With fewer fetuses in the womb, there is more space and nutritional resources for the remaining fetuses. So, even if the toxin has no direct effect on weight, pups of exposed mothers tend to be heavier: the effect of *X* on *Y* is mediated through *N*. If *Y* is regressed on both *X* and *N*, the direct effect of *X* on *Y* is estimated (i.e. the effect not mediated through *N*). If instead the total effect (i.e. the sum of direct and indirect effects) is required, *N* should not be included as a covariate.

Third, suppose that ***X*** is determined before *N* and that *N* is affected by ***X*** but not on the causal pathway from ***X*** to *Y*. If there is an unobserved cluster-constant variable *U* that affects both *N* and *Y*, adjusting for *N* may introduce collider-stratification bias [43]. A first example of this is a dental study in which clusters are mouths, members teeth, *Y* an indicator of presence of dental caries on a tooth, *X* a (cluster-constant) measure of dental hygiene (assumed not to change over time), and *U* is diet. Suppose poor dental hygiene and poor diet both cause tooth loss and, for simplicity, that hygiene and diet are independent. If we regress *Y* on *X* and *N* and look at the parameter associated with *X*, we are comparing patients with good hygiene and a certain number of teeth with patients with poor hygiene and the same number of teeth. More patients in the first group will have poor diet than in the second group. Therefore, diet, which is not a confounder when caries is regressed on hygiene, becomes a confounder when *N* is included as a covariate. A second example is a longitudinal study of aging in which clusters are individuals, *Y*_*j*_ is cognitive function at time *j*, ***X***_*j*_=(1,*j*)^*T*^ consists of intercept and time elements, and *U* is general state of health at the beginning of the study. For individuals who survive to the end of the study, *N* = *M*; for those who die earlier, *N* < *M*. If individuals with worse health at the beginning tend to have faster cognitive decline and higher mortality, then *U* induces ICS. It has been argued that in this setting, *e*_*A*_(***x***) is often a more appropriate target of inference than is inference conditional on *N* [44].

#### 4.1.5. Weighting by *N*^−1^ (weighted independence estimating equations)

When cluster size is informative, either of *e*_*A*_(***x***) and *e*_*T*_(***x***) may be of interest. As stated earlier, when 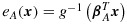, ***β***_*A*_ is consistently estimated by IEE. Williamson *et al.* [11] and Benhin *et al.* [14] showed that when 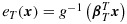, ***β***_*T*_ can be consistently estimated by solving the same IEE but with each cluster's contribution to the estimating equations weighted by *N*^−1^. These are the weighted IEE. Hoffman *et al.* [10] proposed an alternative procedure, which is asymptotically equivalent but more computationally intensive. Chiang *et al.* [45] proposed a more efficient version of weighted IEE, but this makes strong assumptions [46]. Wang *et al.* [47] discussed the use of weighted IEE for three-level data.

#### 4.1.6. Weighting by number with same **X**(type 1 doubly weighted independence estimating equations)

Interpretation of ***β***_*T*_ may be problematic when there is ICS and ***X*** is not cluster-size balanced, because, as noted at the end of Section 2, the association between ***X*** and *Y* may then be confounded by *N*.

Huang and Leroux [15] adapted weighted IEE to deal with CBC caused by unobserved or observed cluster-level confounders (including *N*). They proposed ‘doubly weighted IEE’. Type 1 doubly weighted IEE can be used when ***X*** is categorical and every cluster in the population contains at least one member with each of the possible values of ***X***. Whereas weighted IEE weight each member of the same cluster equally (by *N*^−1^), the weights in type 1 doubly weighted IEE vary within cluster. The inverse weight for member *j* equals the total number of members in the cluster who have ***X*** = ***X***_*j*_. In this way, the total weight given to the members with ***X*** = ***x*** is the same for each possible value ***x*** of ***X***. The purpose here is not to estimate *e*_*T*_(***x***) but rather to describe the association between ***X*** and *Y* in a population of members formed by each cluster in the population contributing one member with each possible value of ***x***. In this population, there is no association between ***X*** and any cluster-constant variable, and hence no CBC. The model 
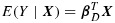
 is assumed to describe the relation between *Y* and ***X*** in this population; solving the type 1 doubly weighted IEE estimates ***β***_*D*_.

It is instructive to consider the relation between ***β*** in Equation (1) and ***β***_*D*_. It is straightforward to show that the non-intercept elements of ***β*** and ***β***_*D*_ are equal when Equation (1) holds, *g* is the identity link and ***X***_rd_=1. When *g* is the identity link but ***X***_rd_≠1, non-intercept elements of ***β***_*D*_ represent a sort of averaged within-cluster effect. When *g* is the logit link and ***X***_rd_=1, the property that population-average effects are less than cluster-specific effects [5] means that the absolute value of each non-intercept element of ***β***_*D*_ is less than or equal to that of the corresponding element of ***β***.

#### 4.1.7. Weighting by propensity score and *N*^−1^ (type 3 double weighted independence estimating equations)

Suppose that interest is in the association between *Y* and a binary treatment. Observed confounders could be handled by including them along with the treatment covariate in ***X*** and using GEE. An alternative to this ‘regression adjustment’ approach is weighting by propensity score [41]. Here, a model is specified for the probability of being treated given the confounders, and then GEE are applied with treatment as a covariate and each treated (respectively, untreated) member inversely weighted by its probability of being treated (respectively, untreated). If this treatment probability model is correctly specified and there is no unmeasured confounding or ICS, a consistent estimate of the marginal treatment effect is obtained. Cepeda *et al.* [48] and Stürmer *et al.* [49] discussed advantages and disadvantages of propensity score weighting compared with regression adjustment.

Just as with the GEE of Section 4.1.2, when A8 is violated, the estimand of the propensity-score weighted GEE depends on the choice of working correlation. In particular, the independence-working correlation yields an estimate of the treatment effect in the population of all members. Huang and Leroux [15] proposed using the independence-working correlation but with additional inverse weighting by cluster size. This method, ‘type 3 doubly weighted IEE’, estimates the treatment effect in the population of typical members of typical clusters. It reduces to what Huang and Leroux [15] called ‘type 2 doubly weighted IEE’ when all confounders are cluster-constant (see also Seaman *et al.* [22] for a discussion of type 2 double weighting).

## 5. Considerations in choosing a method

The choice between cluster-specific and population-average inference has been widely discussed. Neuhaus *et al.* [50] suggested that population-average inference may be more appropriate for cluster-constant covariates, and cluster-specific inference for cluster-varying covariates. Graubard *et al.* [51] agreed that populative-average inference be used for cluster-constant covariates but observed that sometimes it may also be more suitable for cluster-varying covariates. Drum and McCulloch [52] suggested that the choice of approach should depend on whether the analysis is carried out to improve scientific understanding, to make clinical predictions, to make public policy decisions, or for epidemiological purposes. As noted in Section 3.2, the interpretation of ***β***_co_ in a cluster-specific model is problematic when there is CBC or ICS.

Unnecessarily allowing for ICS and/or CBC has an efficiency cost when ICS and CBC are absent. For cluster-specific inference, methods that assume that between-cluster and within-cluster effects of ***X***_va_ are the same are more efficient than those that do not. The gain in efficiency from making this assumption is greatest with binary *Y*, small *N*, and high intra-cluster correlation of ***X*** [31,53]. For example, if *N* = 2 for all clusters, (*X*_1_,*X*_2_) has a bivariate normal distribution with mean 0 and variance 1, and logit *P*(*Y*_*j*_=1∣*X*_*j*_,*u*) = *β*_0_+*β*_1_*X*_*j*_+*u* with *u* ∼ *N*(0,4), *β*_0_=−1 and *β*_1_=0, then the asymptotic relative efficiency of the conditional ML estimator of *β*_1_ versus the ML estimator is 0.67 when *X*_1_ and *X*_2_ are independent, but 0.43 when Corr(*X*_1_,*X*_2_) = 0.5 (Table 1 of [53]). Including as covariates in a GLMM functions of *N* when cluster size is non-informative would be expected to cause some loss of efficiency. However, unless the number of extra parameters introduced to model the effect of *N* is large, this loss may be small. On the other hand, using weighted IEE rather than IEE when cluster size is non-informative may cause substantial loss of efficiency if Var(*N*) is large, unless the intra-cluster correlation of *Y* is also large [14]. For example, if *Y*_1_,…,*Y*_*N*_ are independent and there are no covariates, the relative efficiency of the weighted IEE estimator of *E*(*Y*) relative to the IEE estimator is (from (5.1) and (5.2) in [14]) approximately {*E*(*N*)*E*(*N*^−1^)}xx-xx^−1^, which equals 0.64 when *P*(*N* = 1) = *P*(*N* = 4) = 0.5. Mancl and Leroux [54] (see also [55]) investigated the efficiency loss caused by using IEE rather than GEE with the true working correlation when the true correlation structure is exchangeable. They concluded that when cluster size does not vary, there is no efficiency loss for cluster-constant covariates or for cluster-varying covariates whose mean does not vary between clusters. When cluster size varies, the efficiency loss increases with Var(*N*) and with intra-cluster correlation of *Y*, especially for cluster-constant covariates. For cluster-varying covariates, the efficiency loss is greatest when the covariate vectors of all population members are i.i.d. For example, when *E*(*N*) = SD(*N*) = 20, *X*_1_,…,*X*_*N*_ are i.i.d., Corr(*Y*_*j*_,*Y*_*k*_) = 0.1 for *j* ≠ *k*, and *g* is the identity link, the asymptotic relative efficiency is appproximately 0.7 (Figure 2 of [54]).

So, in the absence of ICS and CBC, greatest efficiency is achieved by using standard GLMM or GEE with a realistic working correlation matrix. Several approaches have been proposed for assessing whether CBC or ICS is present. McCulloch *et al.* [56] recommended testing whether the ML and conditional ML estimators of ***β***_va_ are estimating the same quantity [57]. This is a test for ICS or CBC. When a joint model for *Y* and *N* is used, CBC can be tested for by testing whether the ML estimator of ***β***_vafx_ from the joint model and the conditional ML estimator are estimating the same quantity [58]. Benhin *et al.* [14] presented a formal test of ICS when making population-average inference. This involves estimating ***β***_*A*_ and ***β***_*T*_ using IEE and weighted IEE and testing whether ***β***_*A*_=***β***_*T*_. Alternatively, one could include *N* in a GLMM and test whether its effect is zero. When cluster size is non-informative and ***X***_rd_=1, Ten Have *et al.* [5] pointed out that CBC can cause a deviation from the usual relation between cluster-specific effects and population-average effects. They suggested estimating ***β***_va_ using conditional ML and the corresponding population-average parameter using IEE. In the absence of CBC, the latter parameter is attenuated towards zero compared with the former, with the degree of attenuation a function of Var(*u*). An informal assessment of CBC is therefore to see whether this is approximately true of the estimates. Another strategy to assess CBC would use the poor man's method (or its generalisation in Section 3.3.5) to test for a significant difference between within-cluster and between-cluster effects.

If there is concern about CBC or ICS, standard GLMM/GEE will not be suitable, and another method (or methods) from Section 3 or 4 should be used. For cluster-specific inference, conditional ML has the advantage of making few assumptions, but is limited to canonical link functions. The poor man's method, that of Brumback *et al*., and their generalisation, ‘modelling expectation of random intercept’, also estimate between-cluster or contextual effects, which may themselves be of interest. Unlike conditional ML, however, these methods rely on correct specification of the model for 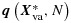. In general, methods making more assumptions would be expected to be more efficient, provided these assumptions are true, and indeed Brumback *et al.* [9] showed that their approach can be more efficient than conditional ML when the model for the expected random intercept is correctly modelled. Conditional ML may therefore be more efficient than conditional GEE when A1 holds, but less efficient otherwise. However, there is a lack of software for conditional GEE, and it does not allow the logit link function. Like modelling expectation of random intercept, treating the random intercept as a fixed effect and joint modelling of *Y* and *N* allow for—and estimate the effects of—cluster-varying covariates with random effects. Treating the random intercept as a fixed effect requires fewer assumptions than the other two methods, but it requires a large average cluster size when *Y* is binary. The other two methods should be more efficient, but require correct specification of 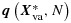 or *f*(*N*∣***X***_co_,***u***). Joint modelling provides an estimate of all of ***β*** and Var(***u***) but requires strong assumptions, including no CBC.

For population-average inference, IEE and weighted IEE are the principal methods for estimating *e*_*A*_(***x***) and *e*_*T*_(***x***), respectively. Hoffman *et al.* [10], Williamson *et al.* [11], and Seaman *et al.* [22] discussed situations where one may be preferred to the other. Marginalising over ***u*** in a GLMM may be more efficient but requires a correctly specified GLMM. Inclusion of *N* as a covariate is one way to handle ICS and may be attractive if there is scientific interest in the effect of *N* on *Y*. However, as discussed in Section 4.1.4, the effect of ***X*** on *Y* not adjusted for *N* may be of more interest. Type 3 doubly weighted IEE is an alternative to weighted IEE for adjusting for observed confounders. It uses propensity score weighting instead of regression adjustment. When there is CBC, IEE and weighted IEE can still be used, but the effects of ***X*** they estimate are confounded. Two methods change the estimand in an effort to describe an unconfounded effect. The poor man's GEE method attempts to eliminate confounding by stratifying clusters according to 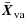 and estimating the within-stratum effect of ***X*** assuming that it is the same in all strata. It allows for ICS when the dependence of *Y* on *N* is through 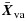. Type 1 doubly weighted IEE eliminate confounding by considering the population formed by sampling one member with each value of ***X*** from each cluster. Its use is limited to situations where ***X*** is categorical and all possible ***X*** values are represented in all clusters (although Huang and Leroux suggested that a mixture of categorical and continuous covariates be handled using weighting for the former and regression adjustment for the latter [15]).

In summary, when choosing a method for analysis, we recommend first deciding whether cluster-specific or marginal inference will best answer the scientific question. When either ICS or CBC is suspected, we may often be concerned about both, and so methods that handle both are recommended in general. It is clear from Table 2 that for marginal inference the choice of method will be driven by the choice of estimand, because none of the methods are excessively computationally challenging. For cluster-specific inference, the next question to be asked (as is apparent from Table I) is whether ***X***_rd_=1 is a reasonable assumption for the data in hand, that is, whether covariate effects are the same in different clusters. If this is assumed, methods such as conditional ML and the poor man's method are relatively simple and well established. For the more general case where covariate effects differ across clusters, the choice of method is more complex and exactly which assumptions best suit the data needs to be carefully considered, following the text earlier in this section and Table I.

## 6. Example

Wood *et al.* [59] examined the association between socio-economic deprivation and preterm neonatal death in Scotland. There were 920 566 births and 440 preterm neonatal deaths during 1992–2008. Deprivation was measured by the Carstairs score, which ranges from 1 (least deprived) to 7 (most deprived). Using IEE, they found a significant population-average association. Treating deprivation as a continuous variable, the crude log OR was 0.600 (SE 0.178) for a six-point increase in deprivation.

An alternative to population-average inference is cluster-specific inference, that is, a comparison of mothers with different deprivation scores attending the same hospital. This might be of interest if, for example, one were contemplating an intervention designed to reduce deprivation (such as offering child-care support to working mothers). The within-cluster (hospital) effect of deprivation could be estimated using a logistic regression model with random intercept for hospital. However, the populations served by different hospitals may differ in their demographic characteristics (in particular, deprivation), and there may be an association between a hospital's quality of care and the mean deprivation of the population it serves. If this is so, there would be CBC and the random-intercept model would be misspecified. We shall investigate whether there is CBC and estimate the within-hospital deprivation effect.

There were 69 hospitals, and the number of births per hospital varied from 19 to 80 749, with mean 13 342. The estimated within-hospital log OR for deprivation from the random-intercept model was 0.546 (SE 0.189) (Table III). The estimated standard error of the random-intercept was 0.249 (*p* = 0.006), indicating evidence for a hospital effect. There was also evidence that the distribution of deprivation varies between hospitals. A proportional-odds model with random intercept (for hospital) was fitted to the deprivation scores. The estimated variance of the random intercepts was 0.498 (*p* < 0.001). Therefore, the conditions required for CBC are present: there is between-cluster variation in the distribution of the covariate and in the distribution of the outcome given the covariate.

**Table III:** Log odds ratios of preterm neonatal death for six-point increase in deprivation score.

Method	log OR	SE	95% CI		*p*
IEE	0.600	0.178	0.251	0.949	0.001
ML estimation of GLMM	0.546	0.189	0.175	0.917	0.004
Conditional ML	0.460	0.195	0.076	0.843	0.019
Poor Man's method					
Within-cluster	0.470	0.198	0.083	0.858	0.017
Between-cluster	1.262	0.641	0.005	2.519	0.049
Model random intercept					
Within-cluster	0.464	0.197	0.079	0.850	0.018
Between-cluster	0.987	0.630	−0.248	2.222	0.117
Cluster size (×1000)	0.007	0.003	0.000	0.013	0.035

Methods are independence estimating equations, maximum likelihood estimate from random-intercept logistic regression model, conditional ML estimate from the same model, poor man's method, and modelling expectation of random intercept as linear function of mean deprivation in cluster and cluster size.

Using conditional logistic regression (conditional ML), the log OR of the within-hospital effect of deprivation on mortality was 0.460 (SE 0.195). As mentioned in Section 5, the fact that this estimate is closer to zero than the population-average estimate of 0.600 is suggestive of CBC [5]. The Tchetgen and Coull test for CBC or ICS mentioned in Section 5 [56,57], which compares the estimates 0.546 and 0.460 from ML and conditional ML, respectively, yielded a *p*-value of 0.07. So, evidence for CBC is not significant but is suggestive. The estimated log OR for the within-hospital effect from the poor man's model was 0.470 (SE 0.198), which is similar to that from conditional ML. The estimated between-hospital log OR was 1.262 (SE 0.641), and the difference between the between-hospital and within-hospital effects was 0.792 (SE 0.676). Although this difference is not significant and we are not here interested in contextual effects, it would be interpreted as meaning that of two women with the same deprivation giving birth in two hospitals with different mean deprivations, the women attending the hospital with the higher mean deprivation would, on average, be at higher risk.

To assess whether ICS was present, we added *N*, the number of women giving birth in each hospital, to the poor man's model 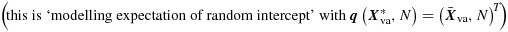. The estimated parameter associated with *N* in this extended poor man's model was 0.00694 per thousand births (SE 0.00329, *p* = 0.04), indicating evidence of ICS. The estimated within-hospital effect of deprivation from conditional ML does not assume non-ICS. However, the presence of ICS raises the possibility that there may be an interaction between cluster-size and deprivation in the model for mortality. However, when we included a cluster-size-deprivation interaction term in the conditional logistic regression model, this interaction term was not significant (*p* = 0.24).

Finally, the ‘treating the random intercept as a fixed effect’ method was used to include a random effect for deprivation. However, we found no evidence that the effect of deprivation on mortality varies between hospitals (*p* = 1.0).

In conclusion, there is weak evidence of CBC (and ICS), with the within-hospital effect of deprivation being possibly smaller than the between-cluster effect. However, accounting for CBC and ICS does not change the substantive conclusion that there is a significant association between deprivation score and mortality.

## 7. Discussion

In this article, we have reviewed methods that have been proposed for population-average or cluster-specific inference in the presence of CBC or ICS. We have clarified what the methods proposed for handling CBC assume about ICS and vice versa. We have explained more fully than previous authors the potential of, and problems with, including cluster size as a covariate. We have proved equivalence of the poor man's method and conditional ML when the identity link is used, in line with an empirical finding of similar estimates in a particular data set noted by previous authors. We found that the methods available typically make strong assumptions about the exact nature of ICS or CBC and about other aspects of the data, and this article has expressed these assumptions in a common format that allows easy comparison. No single method is sufficiently flexible to handle any scenario in which there is ICS or CBC. However, we consider that for most research studies, methods exist which would provide inference of interest.

We hope that our comparison of the assumptions made by the various methods and our discussion of choice of method, and in particular the issue of efficiency, will assist analysts to select suitable methods for clustered data where CBC or ICS is considered possible. As methods to handle CBC or ICS are typically less efficient than standard methods that assume the absence of both, it is likely that some analysts will use a two-stage approach: an initial test for CBC and/or ICS, followed by the selection of a suitable method if either is indicated and a standard method otherwise. Such a strategy is not unreasonable. However, a preferable approach when ICS or CBC is thought possible would be to verify that the results from a standard method do not change substantially when a method designed to handle ICS and/or CBC is used instead. We consider that CBC and ICS are realistic in many research settings and may arise together. We hope this review will lead to a wider awareness of the problems of CBC and ICS and of the failure of standard methods when they are present, and also of the availability of alternative methods.

While we have focused on the estimation of regression parameters in models for uncensored data, other authors have considered ICS in other contexts. Datta and colleagues generalised rank-sum and signed-rank tests to account for ICS [60–62]. Fan and Datta [63] used inverse cluster-size weighting in accelerated failure time models for clustered survival data. VanderWeele *et al.* [64] used an approach similar to the poor man's method for decomposing the indirect effect of vaccination into contagion and infectiousness effects in the presence of spill-over effects.

## APPENDIX

### Appendix A: Conditional ML in standard software

Conditional logistic regression for a binary outcome is widely available in standard statistical software, for example, the *clogit* command in the survival library of R. Some routines for conditional logistic regression can be slow when clusters are large, because they need to evaluate all permutations of *Y*. Fortunately, the *clogit* function uses a clever method to overcome this (see ‘Methods and Formulae’ section of STATA clogit manual).

The conditional ML estimator of ***β*** for the GLMM with a Poisson distributed outcome, log link function and ***X***_rd_=1 is identical to the ML estimator of ***β*** obtained by simply fitting a Poisson regression with a fixed effect for cluster [39].

This is also true when the outcome is instead normally distributed and *g* is the linear link function. However, less computationally intensive methods are available. First, when ***X***_rd_=1, the poor man's method can be used, as it is equivalent to conditional linear regression (Appendix C). Second, Verbeke *et al.* [30] described an even less computationally intensive way to maximise the conditional likelihood of an LMM, which applies whether or not ***X***_rd_=1. Let *q* denote the dimension of ***X***_rd_. For each value of *N* observed in the data set, let ***A***_*N**q*_ denote a *N* × (*N* − *q*) matrix of orthogonal contrasts, that is, a matrix ***A***_*N**q*_ whose columns each sum to zero and for which . Then the conditional ML estimator of ***β*** is equal to the ML estimator of ***β*** from the model that assumes  independently for each cluster *i*. So, standard linear regression can be used.

Verbeke *et al.* [30] also described an extension of this method for fitting conditional LMMs. This involves premultiplying ***Y***^*^ and ***X***^*^ by ***A***_*N*1_ and fitting an LMM for the transformed variables.

Verbeke *et al.* [30] provided sas code for determining the matrix ***A***_*N**q*_. Also, ***A***_*N*1_ can be found using the *contr.poly* command in R.

## APPENDIX

### Appendix B: Proof that A1 is not necessary for consistency of conditional ML in LMM

The contribution of a cluster of size *N* to the conditional likelihood for the LMM with ***X***_rd_=1 is proportional to the density of  given  implied by  [30]. The value , say, to which the conditional ML estimator of ***β*** converges as the number of clusters tends to infinity therefore obeys , regardless of whether or not A1 holds. Let  denote the matrix each of whose rows equals , and let **1** = (1,…,1)^*T*^. The true value ***β***_0_, say, of ***β*** obeys , regardless of whether A1 holds. Because  (because each column of ***A***_*N*1_ sums to zero), it follows that . Therefore, , and so .

## APPENDIX

### Appendix C: Equivalence of poor man's method and conditional ML

We consider the general case where ***X***_co_ and ***X***_va_ can be vectors. Let  denote the matrix each of whose rows equals . For the random-intercept LMM, the contribution of a cluster to the conditional log likelihood of ***Y***^*^ given  is , where *σ*^2^ is the residual error variance [6]. The contribution of the cluster to the score vector for the conditional ML estimate of ***β***_va_ is the derivative of this, that is,

(C1)

The poor man's method generalised to allow ***X***_co_ and ***X***_va_ to be vectors uses the model  with . When *u* is integrated out, the result is a multivariate normal model [65] (Chapter 5). The contribution of a cluster to the log likelihood of this model is , where , with ***J***_*N*_ denoting a *N* × *N* matrix of ones and ***I***_*N*_ denoting a *N* × *N* identity matrix. So, the contribution of the cluster to the score vector for the ML estimate of the poor man's method is

Because , where , and also ,

(C2)

(C3)

Apart from the constant of proportionality  and the replacement of ***β***_va_ by ***γ***_1_, expressions (C.1) and (C.3) are the same. This means that the conditional ML estimate of ***β***_va_ and the ML estimate of ***γ***_1_ are identical, and so are their corresponding estimated standard errors (obtained from the derivative of the score vector).

Also notice that, apart from the constant of proportionality  and the replacement of ***β***_*M*1_ by ***γ***_1_, expression (C.2) is the same as the quasi-score vector used for estimating ***β***_*M*1_ in the poor man's GEE method when the independence working correlation matrix is used. This means that the estimate of ***β***_*M*1_ obtained by applying the poor man's GEE method with independence working correlation is also identical to the conditional ML estimate. However, their estimated standard errors may be different, because the GEE method uses robust standard errors.

Furthermore, notice that expression (C.1) reduces to , which is the same as Goetgeluk and Vansteelandt's [7] conditional GEE equation (3) if ***d*** is given by their equation (8) and Var(***Y***_*i*_∣***L***_*i*_,*C*_*i*_) is assumed to equal  for some . Therefore, for the identity link function, the only difference between conditional GEE and the score equations for the conditional ML estimator is that the latter are based on the assumption that, given ***X***^*^ and ***u***, *Y*_1_,…,*Y*_*N*_ are independent and have equal variance, whereas the former allow *Y*_1_,…,*Y*_*N*_ to be correlated given ***X***^*^ and ***u*** and/or have different variances (and estimate these correlations and variances from the data).
